# Effect of Ketorolac on the Prevention of Postoperative Catheter-Related Bladder Discomfort in Patients Undergoing Robot-Assisted Laparoscopic Radical Prostatectomy: A Randomized, Double-Blinded, Placebo-Controlled Study

**DOI:** 10.3390/jcm8060759

**Published:** 2019-05-29

**Authors:** Jun-Young Park, Jun Hyuk Hong, Jihion Yu, Doo-Hwan Kim, Gi-Ho Koh, Sang-A Lee, Jai-Hyun Hwang, Yu-Gyeong Kong, Young-Kug Kim

**Affiliations:** 1 Department of Anesthesiology and Pain Medicine, Asan Medical Center, University of Ulsan College of Medicine, Seoul 05505, Korea; parkjy@amc.seoul.kr (J.-Y.P.); yujihion@gmail.com (J.Y.); knaaddict@gmail.com (D.-H.K.); foddlcjstk@gmail.com (S.-A.L.); jhhwang11@hotmail.com (J.-H.H.); 2 Department of Urology, Asan Medical Center, University of Ulsan College of Medicine, Seoul 05505, Korea; jhhong@amc.seoul.kr; 3 Department of Anesthesiology and Pain Medicine, Chosun University Hospital, Gwangju 61453, Korea; gramsci1015@gmail.com; 4 Department of Anesthesiology and Pain Medicine, Hangang Sacred Heart Hospital, Hallym University College of Medicine, Seoul 07247, Korea

**Keywords:** catheter-related bladder discomfort, ketorolac, robot-assisted laparoscopic prostatectomy

## Abstract

Urinary catheterization can cause catheter-related bladder discomfort (CRBD). Ketorolac is widely used for pain control. Therefore, we evaluated the effect of ketorolac on the prevention of CRBD in patients undergoing robot-assisted laparoscopic radical prostatectomy (RALP). All patients were randomly allocated to the ketorolac group or the control group. The primary outcome was CRBD above a moderate grade at 0 h postoperatively. CRBD above a moderate grade at 1, 2, and 6 h was also assessed. Postoperative pain, opioid requirement, ketorolac-related complications, patient satisfaction, and hospitalization duration were also assessed. The incidence of CRBD above a moderate grade at 0 h postoperatively was significantly lower in the ketorolac group (21.5% vs. 50.8%, *p* = 0.001) as were those at 1, 2, and 6 h. Pain scores at 0 and 1 h and opioid requirement over 24 h were significantly lower in the ketorolac group, while patient satisfaction scores were significantly higher in the ketorolac group. Ketorolac-related complications and hospitalization duration were not significantly different between the two groups. This study shows ketorolac can reduce postoperative CRBD above a moderate grade and increase patient satisfaction in patients undergoing RALP, suggesting it is a useful option to prevent postoperative CRBD.

## 1. Introduction

Urinary catheterization is generally used during surgery, although it can cause postoperative catheter-related bladder discomfort (CRBD) [1]. CRBD is characterized by discomfort in the suprapubic region, manifesting as urinary urgency and frequency with or without urge incontinence [1,2]. The incidence of CRBD is 47–90% in patients who have undergone elective surgery [3]. CRBD that is above a moderate grade, which is often intolerable and requires treatment [4,5], occurs in 38–57% of patients with a urinary bladder catheter in a post-anesthesia care unit (PACU) [6,7]. Indwelling urinary catheter size and male gender are known independent predictors of CRBD above a moderate grade in the PACU [8]. CRBD can be so disturbing to patients that it is accompanied by postoperative agitation, poor satisfaction of a hospital day, extension of hospital stay, and an increased workload for medical staff [3].

For postoperative pain management, multimodal techniques that include administration of two or more drugs of different mechanisms have been used [9]. Nonsteroidal anti-inflammatory drugs (NSAIDs), cyclooxygenase-2 selective NSAIDs, and opioids should be individualized for the multimodal technique [9]. Opioid and non-opioid analgesics have been administered to 58.9% and 96.5% of patients to manage postoperative pain in general surgery, respectively [10]. However, in contrast to postoperative pain, CRBD has been under-treated and may be resistant to conventional pain management such as opioids because different mechanisms are involved in developing postoperative pain and CRBD [1]. CRBD is induced by involuntary contractions of the urinary bladder mediated by type 3 muscarinic receptor activation [4]. Agents associated with anti-muscarinic effects, such as tolterodine [11], oxybutynin [12], butylscopolamine [2,13], ketamine [4,14], robinul [15], and dexmedetomidine [6], have been used to prevent and manage CRBD, with varying degrees of success. However, these agents are limited because of various adverse effects, including dry mouth, postoperative nausea and vomiting, facial flushing, and blurred vision [1]. 

Ketorolac tromethamine, a NSAID, induces a decrease in prostaglandin levels driven by the inhibition of cyclooxygenase [16]. Ketorolac is widely used as an analgesic in various clinical practices [17,18]. In particular, multimodal analgesia including ketorolac is recommended for effective postoperative pain management [19]. However, the preventive effect of ketorolac on postoperative CRBD has not been studied. Based on these considerations, we hypothesized that ketorolac could prevent CRBD after surgery in male patients requiring urinary catheterization. We evaluated the effect of ketorolac on the prevention of postoperative CRBD above a moderate grade in patients undergoing robot-assisted laparoscopic radical prostatectomy (RALP).

## 2. Materials and Methods

This prospective, randomized, double-blinded, placebo-controlled study was approved by the Institutional Review Board of Asan Medical Center, Seoul, Korea (2018-0749). This study was registered at the Clinical Research Information Service (KCT0003064). 

### 2.1. Patients

Patients aged 20–79 years of age with an American Society of Anesthesiologists physical status ≤2, who were scheduled to undergo elective RALP and voluntarily agreed to this clinical study, were enrolled as participants. Patients with chronic kidney disease, uncontrolled hypertension, morbid obesity, psychiatric disorders, a history of preexisting bladder disease, gastrointestinal ulcers or perforation, hemorrhage, coagulopathy, or asthma were excluded. Those with a history of ketorolac allergy were also excluded. 

### 2.2. Randomization, Concealment, and Blinding

Patients enrolled in the present study were randomized. For the randomization, a web-based randomization software (Random Allocation Software version 1.0, Isfahan University of Medical Sciences, Isfahan, Iran) was used. Randomization was determined with block sizes of four and an allocation ratio of 1:1. Eligible participants were allocated to be given either intravenous ketorolac (ketorolac group) or intravenous normal saline as a placebo (control group) by a computer-generated randomization schedule. 

The randomization codes were enclosed in sequentially numbered, identical, opaque, and sealed envelopes. The envelope was kept in a closed box during the study period. The codes were concealed by the first investigator and were given to the second investigator, who prepared the medications of either ketorolac or normal saline. These medications were prepared in identical syringes with a volume of 2 mL, and labelled with the patients’ names and hospital registration numbers. After the completion of urethrovesical anastomosis during surgery, the medications were administered by the third investigator, who was blinded to the allocation groups. The fourth investigator, who was also blinded to the allocation groups, assessed the outcomes of the study. All other investigators and participants, except the first and second investigators, did not know the group allocation until the data analyses were completed.

### 2.3. Anesthetic and Surgical Techniques

Before surgery, patients were instructed on symptoms of CRBD (i.e., a burning sensation with an urge to void, or discomfort in the suprapubic area). No premedication was administered before the induction of general anesthesia. 

On arrival to the operating room, patient monitoring was performed according to our institutional standards. Intraoperative monitoring included electrocardiography, intra-arterial blood pressure, end-tidal carbon dioxide concentration, and peripheral oxygen saturation. Anesthesia was induced with 5 mg/kg thiopental sodium and target-controlled infusion of remifentanil (Orchestra Base Primea; Fresenius Kabi, Bad Homburg, Germany) with an effect site concentration of 2 ng/mL. Subsequently, 0.6 mg/kg rocuronium bromide was given to facilitate tracheal intubation. Anesthesia was maintained with 2 to 3 vol% sevoflurane and 50% oxygen in medical air. Also, the effect site concentration of remifentanil target-controlled infusion was adjusted to between 2 and 5 ng/mL. The depth of anesthesia was monitored using the bispectral index (A-1050 Monitor; Aspect Medical Systems, Newton, MA, USA), which was maintained between 40 and 60. Systolic blood pressure was maintained at 80–130 mmHg and heart rate was maintained at 60–100 beat/min. Sevoflurane administration and remifentanil target-controlled infusion were intermittently adjusted during surgery according to the bispectral index and hemodynamic parameters. Train-of-four monitoring was used for neuromuscular blockade monitoring. Rocuronium bromide was administered intermittently to maintain train-of-four ≤2 throughout surgery. Mechanical ventilation using an anesthetic machine (Primus; Dräger, Lübeck, Germany) was performed using a fixed tidal volume of 8 mL/kg (ideal body weight) and a respiratory rate of 10–16 frequency/min to maintain an end-tidal carbon dioxide concentration of 35–40 mmHg. A positive end-expiratory pressure of 5 cm H_2_O was applied. The maintenance infusion rate of Plasma Solution A (CJ Pharmaceutical, Seoul, Korea) as a crystalloid fluid was 2–4 mL/kg/h. A colloid fluid was not used during RALP. 

The RALP was performed according to our standard protocols using the da Vinci robot system (Intuitive Surgical, Inc., Sunnyvale, CA, USA). During surgery, all patients were placed in the Trendelenburg position and pneumoperitoneum, which was achieved by continuous carbon dioxide insufflation maintaining an intra-abdominal pressure of 12 cm H_2_O. After skin sterilization for surgery, urinary bladder catheterization was performed with a 16 to 20-Fr Foley catheter after lubrication with lidocaine jelly, and its balloon was inflated with 10 mL of normal saline. To access the space of Retzius, bladder mobilization was performed. A transperitoneal antegrade approach was used to dissect the prostate, and nerve sparing was carried out on all patients on sides that were not suspected for extension of cancer. Pelvis lymph node dissection was performed in intermediate- to high-risk groups as designated by the D’Amico criteria [20]. Urethrovesical anastomosis was performed with a continuous suture. 

Patients were given either ketorolac (30 mg) or an equivalent volume of placebo (0.9% normal saline) intravenously just after urethrovesical anastomosis. At the end of the procedure, sugammadex 2 mg/kg was administered to antagonize residual neuromuscular block after confirming that train-of-four count was ≥2. In the PACU, when the patient reported CRBD above a moderate grade, 50 mg tramadol was administered intravenously as rescue therapy to decrease CRBD as tramadol has a potent antimuscarinic effect [21]. Postoperative pain, defined as sharp pain at the surgical site, was assessed using a numerical rating scale (0 = no pain to 10 = worst imaginable pain). In case of postoperative pain without urgency or suprapubic discomfort like CRBD, 50 μg fentanyl was administered as rescue therapy when pain scores were ≥4 on a numeric rating scale. If the patient complained of both CRBD and postoperative pain, we treated the main chief complaint by either tramadol or fentanyl, and then reassessed the patient.

### 2.4. Assessments

Patient characteristics assessed were age, sex, body mass index, American Society of Anesthesiologists physical status, underlying disease (diabetes mellitus and hypertension), Gleason score, and tumor category. Gleason score was assessed by adding the numeric value of the two most prevalent differentiation patterns on histology obtained during preoperative transrectal ultrasound-guided needle biopsy [22]. Gleason scores were categorized into three groups: under 7, equal to 7, and above 7 [23,24,25]. Tumor category was assessed by digital rectal exam, transrectal ultrasound-guided biopsy, and imaging studies performed preoperatively [26]. Tumor category was classified by the American Joint Committee on Cancer (AJCC) Cancer Staging Manual [27]. Intraoperative variables included operation time, intraoperative fluid administered, and urinary catheter size used.

CRBD above a moderate grade was assessed at 0, 1, 2, and 6 h postoperatively. CRBD above a moderate grade at 0 h postoperatively was assessed just after the patients were transferred to the PACU. The severity of CRBD was considered “mild” when reported by patients only on questioning, “moderate” when reported by patients on their own without questioning and not accompanied by any behavioral response, and “severe” when reported by patients on their own with accompanying behavioral responses, such as flailing limbs, a strong vocal response, or an attempt to remove the catheter [4]. 

Postoperative pain was assessed using a numerical rating scale at 0, 1, 2, and 6 h postoperatively. Doses of all opioids and tramadol administered to patients during the 24 h following surgery were converted to intravenous fentanyl equianalgesic doses according to published conversion factors (intravenous fentanyl 100 μg = intravenous tramadol 100 mg) [28,29]. Patient satisfaction was assessed with a seven-point Likert scale (1 = strongly dissatisfied, 2 = moderately dissatisfied, 3 = slightly dissatisfied, 4 = neutral, 5 = slightly satisfied, 6 = moderately satisfied, 7 = extremely satisfied) [30] at 6 h postoperatively. 

Ketorolac-related complications included acute kidney injury, hemoglobin changes, gastrointestinal bleeding, and desaturation events. Acute kidney injury was assessed by Kidney Disease: Improving Global Outcomes (KDIGO) criteria. According to KDIGO criteria, acute kidney injury is defined as an increase in serum creatinine by 0.3 mg/dL or more within 48 h, or an increase in serum creatinine of 1.5 times or more within the prior 7 days. However, the urine output criterion was not included because of the inconsistency in urine output measurements [5]. Hemoglobin changes, calculated by subtracting preoperative hemoglobin levels from hemoglobin levels at postoperative day 1, were evaluated. Gastrointestinal bleeding during hospitalization was evaluated according to criteria fulfilling one or more of the following conditions: physician-documented frank hematemesis, physician-documented frank melena, heme-positive stool associated with a documented upper gastrointestinal lesion judged to be the source of the bleeding, and active upper gastrointestinal bleeding documented by endoscopy or angiography [31]. Desaturation events, defined as events of saturation below 90%, were assessed in the PACU or general ward until postoperative day 1 [32].

### 2.5. Primary and Secondary Outcomes

The primary outcome was CRBD above a moderate grade at 0 h postoperatively. The secondary outcomes were CRBD levels above a moderate grade at 1, 2, and 6 h postoperatively. Postoperative pain, postoperative opioid requirement, ketorolac-related complications, patient satisfaction, and hospitalization duration were also assessed. 

### 2.6. Statistical Analysis

From the pilot study and our experience, 48% of patients complained of CRBD above a moderate grade at 0 h after RALP. We assumed that ketorolac might decrease the incidence of CRBD above a moderate grade by 50% (i.e., 48% vs. 24%). We calculated that 59 patients would be necessary for each group to acquire statistical significance, with α = 0.05 and β = 0.20. Considering a 10% dropout rate, 66 patients were included in each group. 

The analyses were performed on a modified intention-to-treat basis, which included all randomly assigned participants with all eligible criteria, who performed the study intervention and did not withdraw consent to participate in this study. Data are expressed as mean ± standard deviation, median (interquartile range), or number (%) as appropriate. Normality was assessed using the Kolmogorov–Smirnov test. Categorical variables were compared using the chi-square test or Fisher’s exact test as appropriate. Continuous variables were compared using the independent *t*-test or Mann–Whitney U test as appropriate. The comparisons of postoperative pain scores between the two groups at each time point were analyzed by independent *t*-test and a *p* value < 0.0125 (0.05/4) was considered significant after using Bonferroni correction. Otherwise, a *p* value < 0.05 was considered significant. Statistical analysis was conducted using MedCalc (version 11.3.3.0; MedCalc Software bvba, Mariakerke, Belgium) and SPSS 21 for Windows (version 21.0.0; IBM Corporation, Chicago, IL, USA).

## 3. Results

**Figure 1:**
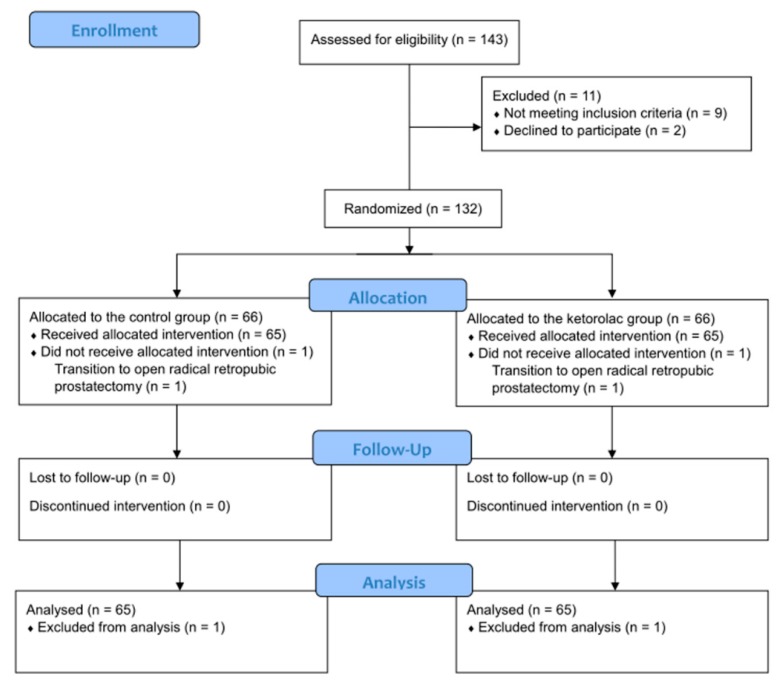
A CONSORT flow chart.

The CONSORT flowchart of this study is presented in Figure 1. During the enrollment process, 143 patients were assessed for eligibility, and 11 patients were excluded. Therefore, a total of 132 patients were included in the present study. Two patients did not receive an intervention, as their surgeries were converted to open prostatectomies. Finally, 130 patients were included in the analysis. All patients were Asian. There were no significant differences in age, gender, body mass index, American Society of Anesthesiologists physical status, underlying disease, Gleason score, tumor category, operation time, intraoperative fluid, and urinary catheter size between the two groups (Table 1). 

**Figure 2:**
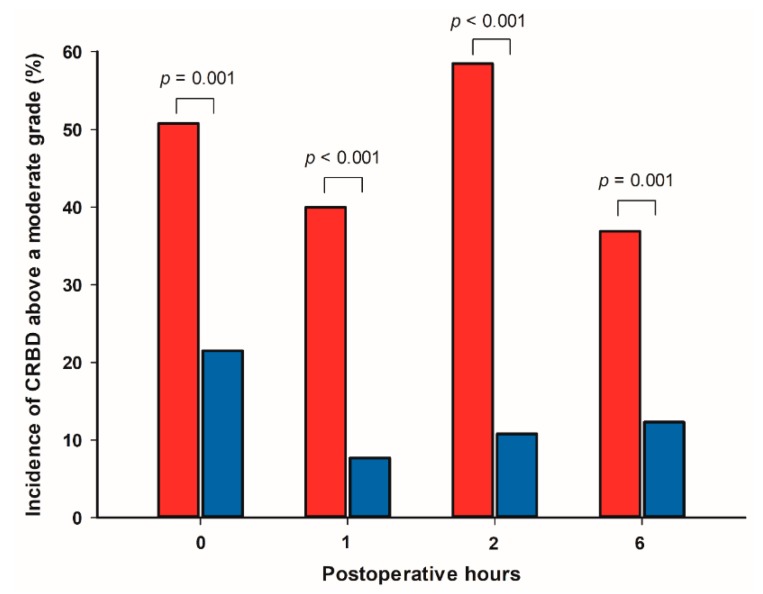
Comparisons of incidences of CRBD above a moderate grade between the control group (red bar) and ketorolac group (blue bar) at 0, 1, 2, and 6 h postoperatively. Each column indicates the incidence of CRBD above a moderate grade. CRBD = catheter-related bladder discomfort; Postoperative hour 0 = upon admission to the postanesthetic care unit.

Incidences of CRBD at 0, 1, 2, and 6 h postoperatively are shown in Table 2. The incidence of CRBD above a moderate grade at 0 h postoperatively was significantly lower in the ketorolac group compared with the control group (14 (21.5%) vs. 33 (50.8%), *p* = 0.001) (Figure 2). In addition, incidences of CRBD above a moderate grade were significantly lower in the ketorolac group compared with the control group at 1, 2, and 6 h postoperatively (5 (7.7%) vs. 26 (40.0%), *p* < 0.001; 7 (10.8%) vs. 38 (58.5%), *p* < 0.001; 8 (12.3%) vs. 24 (36.9%), *p* = 0.001; respectively) (Figure 2). 

**Figure 3:**
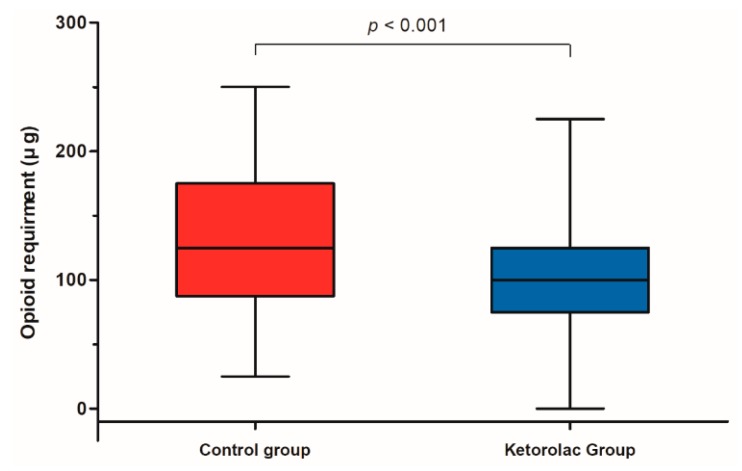
Comparison of opioid requirements within 24 h after surgery between the control group (red box) and ketorolac group (blue box). The line inside the rectangle shows the median. The upper and lower ends of the box indicate the third quartile and first quartile, respectively. Whiskers above and below the box designate 90% and 10%, respectively.

Pain scores at 0 and 1 h postoperatively were significantly lower in the ketorolac group than in the control group (*p* = 0.012, *p* = 0.007, respectively) (Table 3). However, pain scores at 2 and 6 h postoperatively did not significantly differ between the two groups (*p* = 0.766, *p* = 0.132, respectively). Opioid requirement during the 24 h following surgery was significantly lower in the ketorolac group than in the control group (100.0 μg (75.0–125.0) vs. 125.0 μg (87.5–175.0), *p* < 0.001) (Figure 3). There were no significant differences in acute kidney injury, hemoglobin changes, gastrointestinal bleeding, and desaturation events between the two groups (Table 3). Patient satisfaction scores were significantly higher in the ketorolac group than in the control group (5.0 (4.0–6.0) vs. 4.0 (4.0–4.0), *p* < 0.001) (Table 3). There was no significant difference in hospitalization duration between the two groups (7.0 days (5.0–7.0) vs. 7.0 days (5.0–7.0), *p* = 0.722) (Table 3).

## 4. Discussion

In the present study, we found that ketorolac administration significantly decreased the incidences of CRBD above a moderate grade, not only at 0 h postoperatively, but also at 1, 2, and 6 h in male patients undergoing RALP. In addition, pain scores were significantly lower in the ketorolac group than in the control group at 0 and 1 h, but not at 2 and 6 h. The opioid requirement during the 24 h following surgery was significantly lower in the ketorolac group compared with the control group. There were no significant differences in ketorolac-related complications between the two groups. Patient satisfaction scores were significantly higher in the ketorolac group compared with the control group.

Bladder urinary catheterization may induce CRBD after awakening from general anesthesia in the postoperative period [1]. CRBD is a known risk factor for emergence agitation occurring during anesthetic recovery [33]. The incidence of CRBD was higher in the early postoperative period compared with the late postoperative period. Therefore, CRBD is an important issue in the PACU, as patients who receive urinary catheterization usually stay in the PACU for about 1 h postoperatively [15,34,35]. In particular, urgent treatment may be needed for patients experiencing CRBD above a moderate grade, which occurs in 38–57% of patients in the PACU [5,6,7]. Unlike routine perioperative pain management, opioid administration may be regarded as ineffective for CRBD because of the difference between mechanisms of postoperative pain and CRBD [6,34]. For the mechanism of CRBD, the antimuscarinic actions, particularly type 3 muscarinic receptor blockade, are considered to be responsible for the effective management of postoperative CRBD. Therefore, antimuscarinic agents, such as oxybutynin, ketamine, tramadol, and gabapentin, have been evaluated for either treatment or prevention of CRBD [4,21,36]. However, even if such medications are used, the incidence of postoperative CRBD is reported to be high [4,21]. 

In our study, ketorolac effectively reduced the incidence of CRBD above a moderate grade after RALP. Ketorolac, an NSAID and cyclooxygenase inhibitor, inhibits prostaglandin synthesis [37]. Prostaglandins in the bladder, which increase with the occurrence of obstruction, inflammation, and mucosal injury in the urinary bladder [38], induce detrusor muscle contraction. In addition, capsaicin-sensitive C fibers activated by prostaglandins contract the bladder detrusor muscle [39,40,41]. Therefore, ketorolac is thought to reduce CRBD induced by the contraction of the detrusor muscle from the secretion of inflammatory substances and the synthesis of prostaglandins resulting from urinary catheterization. Similarly, paracetamol, which is not an NSAID but is another cyclooxygenase inhibitor, is reported to reduce CRBD scores 0.5, 1, 2, and 6 h after percutaneous nephrolithotomy [42]. However, paracetamol has various adverse effects [42]. Most importantly, paracetamol overdose is a common cause of acute liver failure [43]. Severe liver impairment or hepatic necrosis may occur when the maximum amount of the recommended dose is administered [44,45,46]. In patients with weakened hepatic function, paracetamol can more easily lead to hepatic impairment [44]. Renal impairment not associated with hepatic failure has also been reported [47]. 

We found that ketorolac decreased pain scores at 0 and 1 h postoperatively, but not at 2 and 6 h. This result may be, at least in part, because of the analgesic characteristics of ketorolac. It is known that 30 mg ketorolac is equivalent to 10 mg morphine sulfate or 100 μg fentanyl [28,29]. The peak effect of intravenous ketorolac tromethamine occurs at 75–150 min [18]. In the present study, ketorolac was administered just after the completion of urethrovesical anastomosis, with the surgery ending 30–60 min after ketorolac administration. Therefore, ketorolac administration may influence the postoperative pain score, especially at 0 and 1 h postoperatively. 

NSAIDs such as ketorolac may uncommonly induce renal impairment, peptic ulceration, bleeding diathesis, gastrointestinal bleeding, respiratory distress, and asthma exacerbation [18]. Incidences of these side effects are reported to be 0.17% for renal failure, 1.04% for surgical bleeding, and 0.04% for gastrointestinal bleeding in patients intravenously administered 90 mg ketorolac tromethamine daily for 7 days after surgery [48,49]. Ketorolac is related with an increased risk of gastrointestinal bleeding and renal insufficiency in elderly patients with use exceeding 5 days and higher than 105 mg/day [18]. In the present study, there were no significant differences in acute kidney injury, hemoglobin changes, gastrointestinal bleeding, or desaturation events between the ketorolac group and control group. Therefore, we found that a single administration of 30 mg ketorolac tromethamine does not induce adverse effects associated with NSAID regimens postoperatively. 

Unlike postoperative pain, CRBD might not be managed properly by clinicians. This seems to be partly because clinicians pay less attention to CRBD than postoperative pain. Moreover, conventional analgesics such as opioids may not manage CRBD effectively [1]. In our study, ketorolac reduced postoperative CRBD above a moderate grade and increased patient satisfaction in patients who underwent RALP. In addition, postoperative complications related to ketorolac were not significantly different between the two groups. Therefore, we assume that all patients requiring urinary catheterization can be administered ketorolac for preventing postoperative CRBD when there are no risk factors of side effects associated with NSAIDs, such as renal disease, gastrointestinal ulcers, coagulopathy, and asthma. 

In the present study, patient satisfaction scores were significantly higher in the ketorolac group than in the control group. This may be explained by the reduction of CRBD above a moderate grade within 6 h after RALP in the ketorolac group. In addition, higher patient satisfaction scores are thought to be related to the reduction of pain scores at 0 and 1 h after surgery in the ketorolac group. Therefore, it seems feasible to administer ketorolac to prevent CRBD after RALP.

Our study has several limitations. First, we administered 30 mg ketorolac only once in the present study. Although this single dose of ketorolac had a beneficial effect on the prevention of CRBD above a moderate grade, we did not confirm whether this dose of ketorolac was the optimal dose to prevent CRBD. Therefore, further studies are needed to evaluate optimal doses of ketorolac to prevent CRBD in patients requiring urinary catheterization during surgery. Second, we administered ketorolac just after the completion of urethrovesical anastomosis, and we did not confirm whether this timing of ketorolac administration was optimal to prevent postoperative CRBD. Accordingly, further studies are needed to determine the optimal timing of ketorolac to prevent postoperative CRBD. Third, urinary urgency and urge incontinence are unique characteristics of CRBD, unlike nociceptive postoperative pain. However, it may be difficult to distinguish CRBD and postoperative pain by asking a patient about suprapubic pain after RALP. Therefore, further study will be needed to evaluate the effect of ketorolac on CRBD in patients undergoing surgery in another surgical site.

## 5. Conclusions

Ketorolac reduced the incidence of CRBD above a moderate grade in male patients undergoing RALP. In addition, 30 mg ketorolac administered intravenously during RALP was not associated with any adverse effects. These results suggest that ketorolac administration is an effective and safe option to prevent CRBD above a moderate grade in male patients undergoing RALP who require urinary catheterization.

## Figures and Tables

**Table 1:** Characteristics of study participants.

Variables	Control Group (*n* = 65)	Ketorolac Group (*n* = 65)	*p*-Value
Age (years)	66.7 ± 6.5	65.4 ± 7.0	0.282
Sex (male/female)	65/0 (100/0)	65/0 (100/0)	1.000
BMI (kg/m^2^)	24.5 (23.0–26.6)	24.2 (22.4–25.8)	0.329
ASA PS			0.440
1/2	2/63 (3.1/96.9)	5/60 (7.7/92.3)	
DM	9 (13.8)	9 (13.8)	1.000
Hypertension	27 (41.5)	30 (46.2)	0.596
Gleason score (points)			0.940
≤6	15 (23.1)	16 (24.6)	
7	33 (50.8)	31 (47.7)	
>7	17 (26.2)	18 (27.7)	
T category			0.720
T2	38 (58.5)	40 (61.5)	
T3a	24 (36.9)	18 (27.7)	
T3b	2 (3.1)	6 (4.0)	
T4	1 (1.5)	1 (1.5)	
Operation time (min)	168.0 (156.5–186.5)	167.0 (150.0–184.0)	0.373
Intraoperative fluid (mL)	1100.0 (900.0–1325.0)	1150.0 (800.0–1325.0)	0.393
Urinary catheter size (F)			0.387
16/18/20	32 (49.2)/5 (7.7)/28 (43.1)	30 (46.2)/10 (15.4)/25 (38.5)	

Data are presented as mean ± standard deviation, median (interquartile range), or number (%) as appropriate. BMI = body mass index; ASA PS = American Society of Anesthesiologists physical status; DM = diabetes mellitus; T category = tumor category.

**Table 2:** Postoperative CRBD in patients undergoing robot-assisted laparoscopic radical prostatectomy.

CRBD	Control Group (*n* = 65)	Ketorolac Group (*n* = 65)	*p*-Value
Postoperative hour 0			<0.001
None	12 (18.5)	20 (30.8)	
Mild	20 (30.8)	31 (47.7)	
Moderate	17 (26.2)	14 (21.5)	
Severe	16 (24.6)	0 (0.0)	
Postoperative hour 1			<0.001
None	5 (7.7)	9 (13.8)	
Mild	34 (52.3)	51 (78.5)	
Moderate	25 (38.5)	5 (7.7)	
Severe	1 (1.5)	0 (0.0)	
Postoperative hour 2			<0.001
None	0 (0.0)	1 (1.5)	
Mild	27 (41.5)	57 (87.7)	
Moderate	38 (58.5)	7 (10.8)	
Severe	0 (0.0)	0 (0.0)	
Postoperative hour 6			<0.001
None	0 (0.0)	16 (24.6)	
Mild	41 (63.1)	41 (63.1)	
Moderate	24 (36.9)	8 (12.3)	
Severe	0 (0.0)	0 (0.0)	

Data are presented as number (%). CRBD = catheter-related bladder discomfort; Postoperative hour 0 = upon admission to the postanesthetic care unit.

**Table 3:** Postoperative pain score, opioid requirement, ketorolac-related complications, patient satisfaction score, and hospitalization duration in patients undergoing robot-assisted laparoscopic radical prostatectomy.

Variables	Control Group (*n* = 65)	Ketorolac Group (*n* = 65)	*p*-Value
Postoperative pain score			
Postoperative hour 0	5.4 ± 1.1	4.8 ± 1.5	0.012 *
Postoperative hour 1	3.0 ± 1.3	2.5 ± 0.7	0.007 *
Postoperative hour 2	4.5 ± 1.5	4.4 ± 1.5	0.766
Postoperative hour 6	2.9 ± 1.0	2.7 ± 0.9	0.132
Opioid requirement during 24 h after surgery (μg)	125.0 (87.5–175.0)	100 (75.0–125.0)	<0.001
Ketorolac-related complications			
Acute kidney injury	2 (3.7)	1 (2.2)	>0.999
Hemoglobin changes (mg/dL)	−1.7 ± 1.2	−2.0 ± 1.1	0.148
Gastrointestinal bleeding	0 (0.0)	0 (0.0)	1.000
Desaturation events	1 (1.5)	0 (0.0)	>0.999
Patient satisfaction score	4.0 (4.0–4.0)	5.0 (4.0–6.0)	<0.001
Hospitalization duration (days)	7.0 (5.0–7.0)	7.0 (5.0–7.0)	0.722

Data are presented as mean ± standard deviation, median (interquartile range), or number (%) as appropriate. Hemoglobin change is calculated by subtracting preoperative hemoglobin levels from hemoglobin levels at postoperative day 1. Desaturation events are defined as events of saturation below 90% confirmed in the post-anesthesia care unit or in the general ward until postoperative day 1. Postoperative hour 0 = upon admission to the postanesthetic care unit. * *p* < 0.0125 (Bonferroni-corrected significance level).

## Abbreviations

CRBD	catheter-related bladder discomfort
PACU	post-anesthesia care unit
NSAIDs	nonsteroidal anti-inflammatory drugs
RALP	robot-assisted laparoscopic radical prostatectomy
KDIGO	Kidney Disease: IMPROVING Global Outcomes
SD	standard deviation
IQR	interquartile range
BMI	body mass index
ASA PS	American Society of Anesthesiologists physical status
DM	diabetes mellitus
T category	tumor category
